# Alerting the immune system to DNA damage: micronuclei as mediators

**DOI:** 10.1042/EBC20200016

**Published:** 2020-08-26

**Authors:** Kate M. MacDonald, Soraya Benguerfi, Shane M. Harding

**Affiliations:** 1 Department of Medical Biophysics, University of Toronto, Toronto, ON, Canada; 2 Princess Margaret Cancer Center, University Health Network, Toronto, ON, Canada; 3 Department of Radiation Oncology and Immunology, University of Toronto, Toronto, ON, Canada

**Keywords:** Cell cycle checkpoints, DNA damage, Immune checkpoint blockade, Micronuclei, Radiotherapy

## Abstract

Healthy cells experience thousands of DNA lesions per day during normal cellular metabolism, and ionizing radiation and chemotherapeutic drugs rely on DNA damage to kill cancer cells. In response to such lesions, the DNA damage response (DDR) activates cell-cycle checkpoints, initiates DNA repair mechanisms, or promotes the clearance of irreparable cells. Work over the past decade has revealed broader influences of the DDR, involving inflammatory gene expression following unresolved DNA damage, and immune surveillance of damaged or mutated cells. Subcellular structures called micronuclei, containing broken fragments of DNA or whole chromosomes that have been isolated away from the rest of the genome, are now recognized as one mediator of DDR-associated immune recognition. Micronuclei can initiate pro-inflammatory signaling cascades, or massively degrade to invoke distinct forms of genomic instability. In this mini-review, we aim to provide an overview of the current evidence linking the DDR to activation of the immune response through micronuclei formation, identifying key areas of interest, open questions, and emerging implications.

## Introduction: DNA damage, cell-cycle checkpoints, and micronuclei

In every human cell, every day, tens of thousands DNA lesions are sensed and repaired, owing to a suite of intrinsic programs collectively called the DNA damage response (DDR) [1]. Broadly, the DDR is a cascading set of molecular signals that senses DNA damage, activates cell-cycle checkpoints, repairs the damage, and releases the checkpoint upon damage resolution so that cells can continue to divide [1]. This is, however, not a perfect system. Cells can “pass” checkpoints despite the presence of unresolved lesions, a process known as checkpoint adaptation with potentially dire consequences [2]. A single mitotic error has been shown, over several generations, to culminate in the altered genomic landscape characteristic of adult cancers [3–5]. Recent work has expanded our understanding of the role of the DDR, demonstrating that through several mechanisms it can alert the immune system to ongoing DNA damage, promoting immunological recognition and elimination of genetically unstable cells.

The precise molecular cascade initiated by DNA damage is highly specific to the type of DNA lesions that triggered it (double-stranded breaks [DSBs] vs. chemically altered bases, for instance) [1], and is influenced by factors such as cell type, genetic background, and phase of the cell cycle [2,6–8]. There are DSB-induced cell-cycle checkpoints within S phase and at the G1/S and G2/M transitions. All of these checkpoints serve in part to avoid replicating or entering mitosis with damaged DNA, and each checkpoint employs a specific signaling program for initiating, maintaining, and eventually reversing the checkpoint. Each of the DSB-induced cell-cycle checkpoints are initiated by the ataxia telangiectasia mutated (ATM) and/or ATM-related (ATR) kinases (reviewed in depth by [2]). The conventional understanding is that when DNA damage is repaired, ATM and ATR signaling stops, and the checkpoint actively dissolves in part through the activity of phosphatases and deubiquitylating enzymes, allowing cell cycle re-entry [2]. However, multiple lines of evidence from yeast to humans indicate that cell-cycle checkpoints are not so dichotomous [9]. Instead, checkpoints appear to be engaged and released at certain DSB thresholds, and at certain magnitudes of ATM/ATR signaling, which circumvents the checkpoint and allows cell-cycle progression despite the presence of unresolved DNA lesions [2,9,10]. Cells entering mitosis with DNA damage are especially vulnerable to the generation of gross chromosomal abnormalities [4,5], and DSB repair is actively suppressed during M phase to counter amplification of chromosomal instability [11,12]. Cancer cells may leverage imperfect checkpoint signaling to move through mitosis with abnormal karyotypes or high mutational burdens, but to date the reasons for checkpoint adaptation in otherwise healthy cells are not entirely clear [13]. In yeast, checkpoint adaptation not only leads to genomic instability, but also appears to facilitate repair of the damage by mechanisms active in cell-cycle phases after the one in which damage was initially incurred, thus maximizing survival [14]. In multicellular organisms, checkpoint adaptation may promote mitotically driven cell death, which could serve to eliminate irreparably damaged cells, especially in cell types not prone to undergo apoptosis [9]. Similarly, recent evidence suggests that traversal of mitosis with DNA damage can trigger post-mitotic inflammatory signaling and immune-mediated elimination of the damaged cell(s), limiting genomic instability and tumorigenesis [15,16].

One potential fate for cells surviving checkpoint adaptation with unresolved DNA damage is the formation of micronuclei. Micronuclei contain either acentric chromosomal fragments or whole lagging chromosomes, depending on the type of insult to which the cell has been exposed. For example, when cells fail to repair DSBs, often the most lethal lesion caused by ionizing radiation (IR) [17], they generate untethered, acentric pieces of the genome [18,19] while exposure to other genotoxic agents, including mitotic spindle poisons such as paclitaxel or nocodazole, cause whole chromosome segregation errors [5,20,21]. At mitosis, these acentric fragments or whole lagging chromosomes are left behind at the metaphase plate as the rest of the genome segregates, and they are not incorporated within the newly forming nuclear envelopes at mitotic exit [18,22,23]. Instead, they are sequestered into micronuclei, a fragment of double-stranded DNA encased in a version of a nuclear envelope, residing in the cytosol at interphase and distinct from the primary nucleus [19,24]. Micronuclei have emerged as important features of and functional entities in cells that have experienced DNA damage, in at least two major ways: directly, when micronuclear envelope rupture exposes double-stranded DNA to the cytosol, where it is recognized by viral pattern recognition receptors (PRRs) and invokes an inflammatory signaling program [16,25–27]; and indirectly, as micronucleation is one of the initiating events in a cascade of accumulating genomic instability, potentially leading to the generation of neoantigens implicated in cancer immunoediting [4,5,21]. Micronuclei generation following unresolved DNA damage therefore acts as a mediator between the DDR and immune recognition, and this mini-review will highlight several mechanisms through which DNA damage-immune system crosstalk can be achieved.

## Immune recognition of DNA damage in the clinic: radiation therapy and immunoncology

IR has been used to treat cancer since 1896, and nearly half of all cancer patients today receive radiotherapy (RT) as part of their treatment [28]. RT can directly cause the DNA damage-driven clonogenic death of carcinoma cells, often by mitotic catastrophe [29,30]. Seminal observations have shown that radiation therapy alone could not control tumor growth in mice that had their CD8+ T cells depleted [31], indicating the immune system may also play an important role during RT for human cancers. This is one conceptual basis for the clinical combination of radiotherapy with immune checkpoint blockade (ICB): a reactivation of suppressed lymphocyte activity, so the immune-stimulating properties of RT-induced DNA damage can be fully realized (reviewed in depth by [32]). Systemic anti-tumor immune responses have been of clinical interest since the abscopal effect was presented by RH Mole in 1953, describing a phenomenon whereby distal tumors regress outside the irradiated field [33]. Abscopal effects are rarely observed clinically, but an immune-mediated systemic response to localized therapy has become a key focus of efforts to understand RT-ICB synergy [34]. RT-ICB has seen successes in clinical use [35–38] but as studies continue, it is clear that considerable gaps remain in our understanding of DNA damage-immune interactions, and their role at both a local and systemic level. Further work is necessary to fully understand the immune-mediated responses to DNA damage, and whether this is practical to realize in a clinical setting when treating a single lesion in metastatic disease [39]. In the remainder of this review, we will highlight the specific cell-intrinsic mechanisms that connect DNA damage to immune activity, which have the potential to be harnessed for clinical use.

## Micronuclei are a reservoir of immunostimulatory nucleic acids

**Figure 1:**
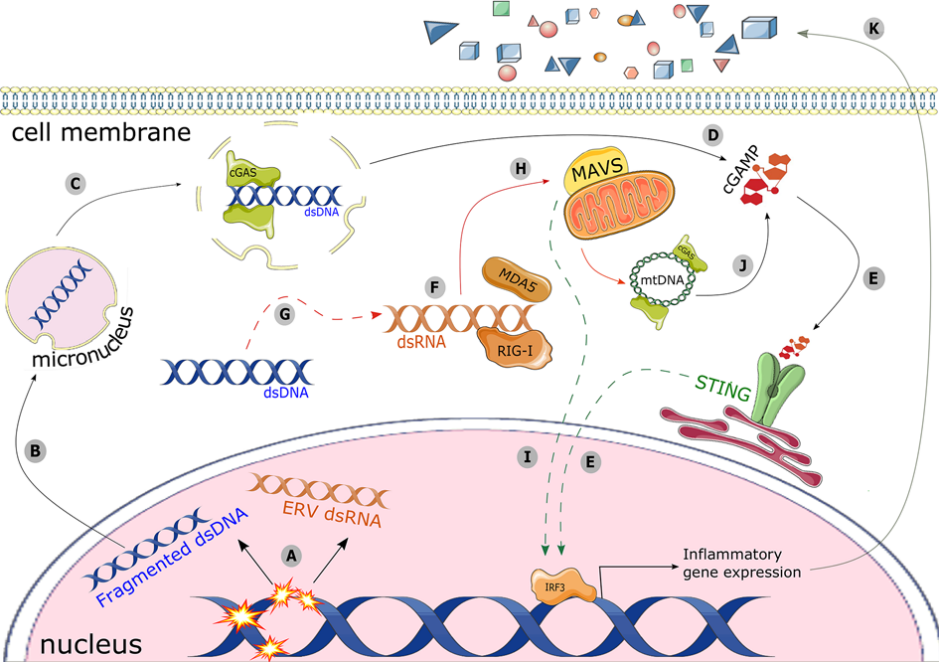
Inflammatory signaling following DNA damage can be achieved via several cytosolic nucleic acid recognition pathways (**A**) DNA damage in the nucleus causes dsDNA fragments to become separated from the rest of the genome, and can cause the aberrant expression of endogenous retroviral elements (ERVs) as dsRNA. (**B**) dsDNA fragments can be sequestered into micronuclei, residing in the cytoplasm. (**C**) Micronuclear envelopes are prone to rupture, whereupon cGAS can recognize the enclosed dsDNA, (**D**) produce cGAMP, and (**E**) signal through STING to promote inflammatory gene expression. (**F**) dsRNA in the cytosol, which may come from ERV dsRNA or from (**G**) transcribed dsDNA, is recognized by RLRs such as RIG-I and MDA5. (**H**) This alerts mitochondrion-bound MAVS, (**I**) leading to expression of inflammatory genes. (**J**) Mitochondrial DNA is another cGAS ligand, and will also initiate STING-driven inflammatory signaling. (**K**) Ultimately, inflammatory gene expression induced through any of these pathways can result in extracellular release of cytokines such as type I interferons, allowing a damaged cell to influence its microenvironment.

One mechanism linking the DDR to innate immune activation is the release of endogenous, immunostimulatory nucleic acids into the cytoplasm. Up-regulated inflammatory signaling is a well-known consequence of IR exposure [40], and in 2017 several groups showed that this DNA damage-induced signature depends on the accumulation of endogenous DNA fragments in the cytosol, including within micronuclei [16,25–27]. Damage-induced micronuclear fragments recruit their own nuclear envelope at mitotic exit, but the process by which this occurs is not well understood, and compared with the primary nucleus it appears to be largely defective [22,23]. Micronuclear envelopes are structurally fragile, tending to rupture in interphase and exposing double-stranded DNA (dsDNA) to the cytosol [20,22]. Here, dsDNA is recognized by viral PRRs specific for this ligand such as cyclic GMP-AMP synthase (cGAS) [16,25–27]. cGAS generates cGAMP, a ligand for ER-bound STING (stimulator of interferon genes) and STING activates the transcription factors IRF3 and NF-κB, leading to induction of a suite of inflammatory genes and cytokine secretion [16,41–43]. These secreted cytokines can act in an autocrine and paracrine fashion, for example through recognition of IFN-β by type I interferon receptors, which signal to JAK/STAT to further up-regulate interferon-stimulated genes (reviewed by [44]). An inflammatory signaling cascade is thus a possible downstream consequence of unresolved DNA damage, but negative regulators will balance this response. For example, the TREX1 3′ exonuclease may localize to ruptured micronuclei, degrading the exposed dsDNA and obviating recognition by cGAS [45]. Some micronuclei are removed from the cytosol by autophagy, similarly preventing PRR nucleation [46]. The specific circumstances under which this degradation might occur remain to be fully understood, but prior work suggests that TREX1 induction relies on a particular threshold of DNA damage [3,47], and these observations indicate that cGAS-STING-mediated inflammatory signaling is not an inevitable outcome of micronuclei formation or rupture. Collectively, these innate viral response pathways have the potential to alert the immune system following cell-intrinsic double-stranded breaks and micronucleation. Through pattern recognition receptors, micronuclei are a nexus for the interface between DNA damage and the tumor immune microenvironment (Figure 1A–E).

Tumors with a high burden of DDR gene mutations tend to be those with the greatest increase in cytosolic dsDNA-driven inflammatory signaling, and inhibiting DDR proteins such as ATR potentiates this response [48,49]. That said, should major unresolved DNA damage events cause cell death by mitotic catastrophe or other means rather than survival and micronucleation, this would preclude dsDNA release and a prolonged inflammatory response. Additionally, the ability of a cell to bypass a checkpoint, necessary for micronuclei formation, probably depends on the actual burden of DNA damage [50–52]. Inhibition of non-homologous end joining (NHEJ), which maintains a high load of DSBs post-IR, stops cells from progressing into mitosis and initiating micronucleus-associated inflammatory signaling, and prevented an abscopal response with RT-ICB in a murine melanoma model [16]. While the G2/M checkpoint has been experimentally implicated in this process, checkpoints at other phases in the cell cycle are also likely to interfere with micronucleation. Future work is necessary to fill these gaps in our understanding of DNA damage burden, cell-cycle checkpoint engagement, and downstream inflammatory responses, particularly as they relate to micronuclei formation and cytosolic dsDNA sensing.

## Other sources of immunostimulatory nucleic acids

Nuclear DNA, exposed to the cytosol via micronuclei, is not the only potential source of immunostimulatory dsDNA. Exosomes are extracellular vesicles that contain protein, lipids, DNA and/or RNA, and they may play a role in endogenous DNA exposure via a paracrine signaling-like mechanism. Immunostimulatory dsDNA or cGAMP can be packaged into exosomes and released from damaged cells, where they are picked up by immune or other cell types to initiate STING signaling [53–57]. Ionizing radiation is also known to cause mitochondrial damage, and failure to repair this damage leads to the release of mitochondrial DNA (mtDNA) which, similar to bacterial genomes, is extensively hypomethylated [58,59]. Like micronuclear DNA, mtDNA is recognized by cGAS to drive STING-mediated inflammatory signaling [58] (Figure 1J). Disrupted mitochondria can also trigger a JNK-driven retrograde signaling cascade to block the activity of DDR protein 53BP1 in the primary nucleus, permitting extensive end resection at nuclear DSBs and the cytosolic accumulation of chromatin fragments [60]. Furthermore, mitochondrial damage can intersect DNA repair and inflammatory cascades with programmed cell death, through p53. P53 is an essential gatekeeper of the G1/S cell-cycle checkpoint, and is also responsible for executing apoptosis via the intrinsic mitochondrial pathway [61,62]. However, given that apoptosis is generally considered an immunologically silent event (reviewed by [63]), the influence of mtDNA on DDR-mediated inflammatory programs may not directly follow from intrinsic apoptotic signaling. Primary nuclear envelope rupture, for example via chromatin bridge breakage, is yet another mechanism whereby dsDNA can become exposed to the cytosol [3,4,45]. DSBs can accumulate in the primary nucleus if these ruptures are not immediately repaired, which primes micronuclei formation in subsequent mitoses [64]. Understanding the specific influences that result in death over damage repair in both the primary nucleus and mitochondria has long been an area of active research, and future studies will expand our understanding of their roles in DDR-driven immune recognition or indolence.

Immunostimulatory RNA fragments can also be generated as a consequence of unresolved DNA damage. Acute DNA damage can cause the aberrant expression of short interspersed elements (SINEs) [65] and endogenous retroviruses (ERVs) [66,67], which can persist in the cytosol in double-stranded RNA (dsRNA) form. Though the specific mechanism of ERV activation is not always clear, epigenetic modifications can de-repress these loci, and there is currently great interest in applying DNA de-methylating drugs for immune activation in cancer [68]. ERV dsRNA accumulates in the cytosol where it is recognized by RIG-I-like receptors (RLRs), a set of RNA-sensing PRRs that includes RIG-I, MDA5, and LGP2 [69,70]. RIG-I and MDA5 alert the RLR adaptor protein MAVS, promoting a type I interferon response post-radiotherapy and a broadly immunostimulatory phenotype [71] (Figure 1F–I). Not all nucleic acid sensing pathways are straightforwardly immunostimulatory, however, as LGP2 has been shown to negatively regulate the interferon response and lead to an immune-tolerant phenotype following IR-induced DNA damage [69]. Differing microenvironmental effects must also be considered, as cancer cell-secreted interferon-stimulated genes have been implicated in tumor radioresistance and resistance to immunotherapy [72,73]. Further work is needed to fully understand the contexts in which nucleic acid sensing following DNA damage can invoke an immunostimulatory or immunosuppressive response, and its role in the outcome of cancer therapy.

The DNA and RNA sensors of cytoplasmic nucleic acids trigger molecular signaling cascades with substantial overlap, and while each nucleic acid ligand has a canonical PRR and downstream effect on gene expression, there likely exists considerable cross-talk between these pathways. A few examples of this have been recently reported: STING, downstream of dsDNA-activated cGAS, can also be stimulated by the RNA-sensing RLRs [74]. Immunostimulatory dsDNA can be transcribed into dsRNA, and recognized by MAVS [75] (Figure 1G). Another PRR, ZBP1, recognizes both dsDNA and dsRNA in their left-handed Z-form [76,77]. Its activity promotes assembly of the NLRP3 inflammasome, a multiprotein construct that initiates a pro-inflammatory response upon recognition of its target [78]. STING, too, can activate NLRP3 directly [79,80] and dsDNA is recognized by another inflammasome, AIM2 [81]. AIM2 activates caspases 1 and 3, which negatively regulate interferon signaling by cleaving cGAS, MAVS, and IRF3 downstream of STING [82,83]. It is possible that micronuclei may be recognized by dsDNA-specific PRRs other than cGAS and/or engage pathways outside of cGAS-STING, including PRRs recognizing dsRNA [15]. The ultimately pro- or anti-inflammatory response established following DNA damage will likely depend on the balance of responses exerted by the aforementioned pathways, the influence of cell type and microenvironment, and may even depend specifically on the type of DNA damage to which the cell is responding. The relative contributions of each of these pathways in different cancer types, following RT or various chemotherapeutic regimens, and the frequency with which they co-occur in a given cell is currently not known, but will almost certainly influence any immune-modulating outcome of the DNA damage response.

## Micronuclei-initiated genomic instability

**Figure 2:**
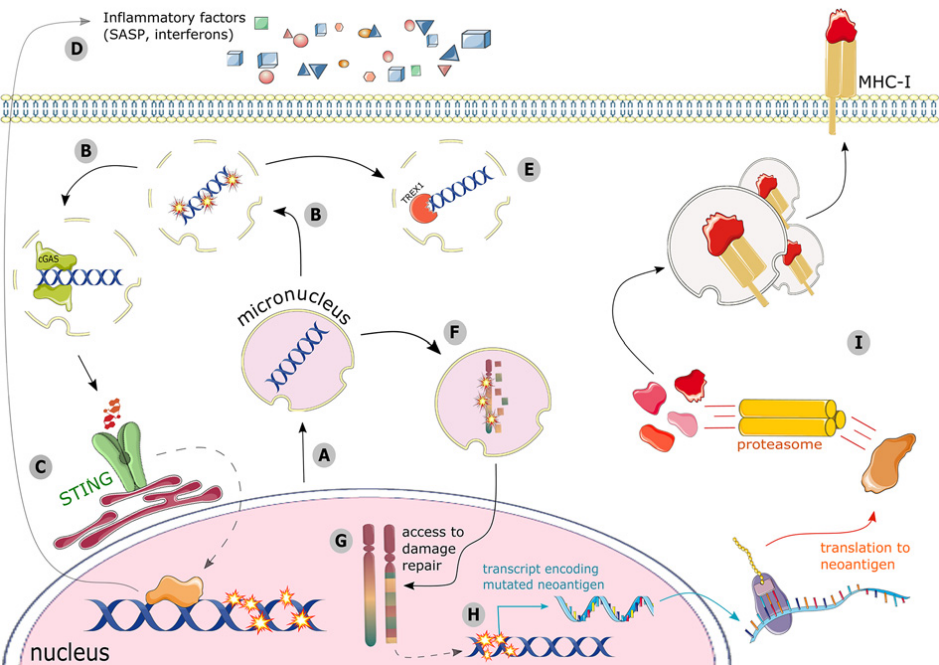
DNA damage can signal the immune system via multiple, intersecting mechanisms (**A**) DNA damage and checkpoint adaptation leads to micronuclei formation. (**B**) Micronuclear envelopes are prone to rupture in interphase, rendering them vulnerable to DNA damage and to recognition by cGAS. (**C**) cGAS nucleation drives an inflammatory response through STING signaling, (**D**) culminating in the release of inflammatory cytokines and/or SASP factors into the microenvironment. (**E**) Ruptured micronuclei are also exposed to cytosolic nucleases such as TREX1, whereupon the dsDNA within the micronucleus can be degraded, precluding cGAS recognition and inflammatory signaling. (**F**) Prior to their envelope rupturing, micronuclei may try to replicate their DNA. This leads to the accumulation of DNA damage and a fragmented, under-replicated micronuclear genome. (**G**) When micronuclear chromosomes are re-incorporated into the primary nucleus, these damaged fragments can be re-ligated into disarranged blocks, driving major genomic instability events such as chromothripsis. This outcome is not mutually exclusive with the models depicted in (**B-E**), as DNA damage via non-replication-associated means can accrue in ruptured micronuclei, and exposed dsDNA can be re-incorporated into the primary nucleus. (**H**) Ongoing genomic instability can deposit mutations in protein-coding genes, generating neoantigens. (**I**) Neoantigen proteins are degraded by the proteasome, and peptide fragments are loaded onto MHC-I, where they are presented for recognition by CD8+ T cells.

Prior to micronuclear envelope rupture, micronuclei are generally capable of primary nuclear activities including transcription [23,84,85], DNA damage repair [23,86–88], and DNA replication [21,23,89]. Micronuclear replication in particular has linked micronuclei to a major genomic instability event called chromothripsis [4,5,21,23]. Some micronuclei attempt to replicate their DNA alongside the primary nucleus, but this process is largely defective, leading to under-replicated regions of the micronuclear genome and their accumulation of extensive DNA damage [4,5,21,23]. At the next mitosis, some cells attempt to re-incorporate the fragment contained within the micronucleus back into the primary nucleus [21]. The fragmented micronuclear genome gains access to the appropriate repair machinery in the primary nucleus, and it can be re-ligated in disarranged blocks, giving rise to the extensive rearrangement along a whole chromosome, single chromosomal arm, or even multiple chromosomes that is the distinguishing feature of chromothripsis [5,21] (Figure 2F,G). Chromothripsis is thought to be an initiating event in cancer development, and regions of the cancer genome carrying evidence of chromothripsis often coincide with driver mutations [90–92]. Over several cell division cycles these degenerative processes initiated in micronuclei can result in the highly complex, chromothriptic genomes characteristic of some adult tumors [4]. Rather than a passive indicator of past DNA-damaging events, micronuclei are now known to be active instigators of the chromosomal instability that is a hallmark of cancer.

When their envelopes rupture, DNA replication and repair within micronuclei stop [22,23]. However, ruptured micronuclei can still be re-incorporated into the primary nucleus and show signs of chromothripsis [5], likely indicating that aberrant micronuclear replication is not the only source of damage that can promote genomic instability. Despite disrupted DNA replication, micronuclear rupture leads to increased DNA damage as measured by γH2AX [23]. TREX1 activity has been implicated in the onset of chromothripsis, but its role in micronuclear DNA damage is still the subject of active study [4,93]. The consequences of micronuclear envelope rupture, its impact in accumulating genomic instability, and its recognition by both DDR proteins and PRRs are still emerging. Finally, while this discussion has primarily focused on the outcome of DSBs, particularly applicable to RT, micronuclei formation has been reported following cellular exposure to chemotherapeutics with other kinds of DNA-damaging effects. These include chronic replication stress [84,88], transcription stress [94], or alkylating base damage [95]. It will be important to understand the factors that govern micronuclei generation and activity under these divergent circumstances, given the influence that micronuclei can have in genomic instability and immune recognition of damaged cells.

## Genomic instability is recognized by the immune system

Genomic instability can be detected by the immune system in several ways. Accumulating instability following aneuploidy (which can occur when whole chromosomes are segregated into micronuclei [5,21,96]) has been shown to directly up-regulate expression of natural killer cell ligands and the senescence-associated secretory phenotype (SASP), recruiting innate immune cells [96]. The SASP, typically defined as a pro-inflammatory secretome, was one of the first comprehensively described processes of IR-induced cytokine secretion [97], and a large subset of SASP cytokines are dependent on DDR signaling for their production [98]. Because of this, the SASP can be considered an extracellular extension of the DDR that influences the microenvironment through paracrine signaling [99] (Figure 2D). Irradiated and extensively damaged cancer cells experiencing a persistent DDR and initiating the pro-inflammatory SASP can recruit immune cells for tumor clearance [100,101], but the SASP can also promote tumor-sustaining inflammation or a stromal environment that supports cancer growth and immune evasion [102,103]. Indeed, chronic signaling through cGAS-STING appears to promote senescent phenotypes and can drive aneuploid tumors towards metastasis [25,104,105]. The specific factors released with the SASP can vary, depending on the cell type and whether senescence was initiated by specific oncogenes, telomere attrition, or DNA damage [97,106]. Understanding the role of the DDR in modulating these extracellular communications could uncover potential therapeutic targets favoring anti-tumor immunity, and lead to a better appreciation for their role in carcinogenesis [99].

Ineffective repair of DNA damage can increase mutational burden and chromothripsis-associated chromosomal alterations. This genomic instability can subsequently lead to the production of neoantigens, which are altered forms of self-peptides that appear foreign to the immune system [107]. Genomic instability in general [108–110] and mutations to DDR proteins in particular [111,112] are a positive predictive biomarker for ICB efficacy. Together, these observations indicate that a high mutational burden leads to elevated neoantigen production, promoting the immunological recognition and infiltration necessary for effective ICB treatment. For novel neoantigens to exert any influence over tumor cell clearance through adaptive immunity, they must be presented on the cell surface by major histocompatibility complex class I (MHC-I), and this process has also been linked to the DNA damage response. Unchecked, broad-scale DNA damage expands intracellular peptide pools for loading onto MHC-I [113], up-regulates MHC-I expression [48,113,114], and promotes the cell’s elimination by CD8+ T lymphocytes, particularly when combined with ICBs [114–117] (Figure 2H,I). Thus, DNA damage during cancer therapy is correlated with increased neoantigen burden and immune recognition, offering one potential mechanistic underpinning for synergy between DDR inhibitors or radiotherapy and ICB. Similarly, it is likely that emerging genomic instability during carcinogenesis acts as a selective pressure to suppress immune-mediated tumor clearance, for example by activating immune checkpoints. The balance between genomic instability, tumor immune-mediated clearance, and the cytokine milieu of the tumor microenvironment is critical to understanding both tumorigenesis and treatment responses, and forms the basis of many ongoing studies.

## Conclusion

In a cell that slips cell-cycle checkpoints and moves through mitosis, unresolved DNA damage can compound over generations of cell division into highly complex, unstable genomes, a hallmark of cancer development [4]. Checkpoint adaptation generates micronuclei [24], subcellular structures that allow the cell to broadcast ongoing genomic instability to the immune system, via PRR-induced inflammatory signaling [16,27] or an exacerbated mutational burden that can promote neoantigen formation or presentation [5,21]. In a clinical setting, there is great interest in exploiting communication between DNA damage and the immune system for the treatment of cancer by combining DNA-damaging therapies (RT or chemotherapy) and ICB, and micronuclei have been implicated in this synergistic process [16]. An improved collective understanding of micronuclear biology, development of genomic instability, and the communication between multiple cell types in the tumor microenvironment is critical to understanding carcinogenesis. This work will also contribute to the rational adaptation of these concepts toward optimal therapeutic implementation, combining DNA-damaging agents and immunotherapy in the cancer clinic.

## Summary

- Cell-cycle checkpoint failure, adaptation, or bypass allows the cell to proceed though mitosis despite the continued presence of DNA damage. This process can generate micronuclei.
- Micronuclei are recognized by viral pattern recognition receptors that initiate inflammatory gene expression, alerting the immune system to the presence of damaged or mutated cells.
- Major genomic instability events such as chromothripsis can take place within micronuclei, which may indirectly alert the immune system to DNA damage through neoantigen formation.
- The source and recognition of DNA or RNA species, either in the cytoplasm or micronuclei, is an emerging feature of the DNA damage response and has important implications for cancer development and therapy.

## Abbreviations

AIM2: absent in melanoma 2
cGAS: cyclic GMP-AMP synthase
DDR: DNA damage response
DSB: double-stranded break
dsRNA: double-stranded RNA
ERV: endogenous retrovirus
ICB: immune checkpoint blockade
IR: ionizing radiation
IRF3: interferon-regulatory transcription factor 3
MAVS: mitochondrial antiviral signaling protein
MDA5: melanoma differentiation-associated protein 5
MHC: major histocompatibility complex
NF-κB: nuclear factor κ light chain-enhancer of activated B cells
NLRP3: NOD-, LRR- and pyrin domain-containing protein 3
PRR: pattern recognition receptor
RIG-I: retinoic acid-inducible gene I
RT: radiotherapy
SASP: senescence-associated secretory phenotype
STING: stimulator of interferon genes
TREX1: three-prime repair exonuclease 1
ZBP1: Z-DNA-binding protein 1
